# Family Members’ Experiences of a Person-Centered Information and Communication Technology–Supported Intervention for Stroke Rehabilitation (F@ce 2.0): Qualitative Analysis

**DOI:** 10.2196/69878

**Published:** 2025-05-02

**Authors:** Gunilla Eriksson, Kajsa Söderhielm, Malin Erneby, Susanne Guidetti

**Affiliations:** 1 Division of Occupational Therapy, Neurobiology, Care Sciences and Society, Karolinska Institutet, Alfred Nobels Allé 23, Huddinge, 14183, Sweden, +46 852480000; 2 Landstinget Sörmland, Nyköping, Sweden

**Keywords:** significant others, informal caregivers, SMS, smartphone, goal setting, eHealth, digital health, telehealth, interdisciplinary rehabilitation, occupational therapy

## Abstract

**Background:**

Stroke often leads to long-term effects on daily activities and participation. Consequences impact not only stroke survivors but also their close networks, and capturing their experiences is crucial for the development of effective interventions. F@ce 2.0 is a person-centered, information and communication technology (ICT)–supported stroke rehabilitation intervention currently being evaluated.

**Objective:**

This study aims to describe family members’ experiences of the F@ce 2.0 intervention from the perspective of being a caregiver to a stroke survivor.

**Methods:**

Participants were family members (n=7) of stroke survivors participating in the intervention. Semistructured interviews were conducted at 2 time points, postintervention and 6 months postbaseline, resulting in a total of 13 interviews. Data was analyzed using qualitative inductive content analysis.

**Results:**

An overarching theme was developed from 4 categories. The main theme was the potential of F@ce 2.0 as a support for family members of stroke survivors in the sudden change of life. The categories were: dialogue and partnership with the F@ce 2.0 team, resuming daily activities lowers the demand for family support, support and involvement through the ICT component of F@ce 2.0, and engagement in F@ce 2.0, leading to suggestions for development.

**Conclusions:**

This study aligns with previous research delineating the effects of stroke on family members of stroke survivors. Participants highlighted the positive impact of the focus on daily activities within the intervention. Furthermore, the ICT component was perceived as a support in structuring rehabilitation. Participants, however, suggested further development, both in terms of content and technology.

## Introduction

### Background

Stroke is a major noncommunicable disease and a leading cause of long-term disability globally [1,2]. In Sweden, around 25,000 people have a stroke each year [3]. Stroke can lead to significant changes in participation in daily activities [4], work roles [5], and social life [6]. These negative consequences in everyday life affect not only the stroke survivor but also the close network. A need for rehabilitation teams to rethink who is considered a part of this network has been suggested [7]. In this study, the focus is, however, on family members, since all participants were either spouses or children of stroke survivors.

Being a family member to a stroke survivor has been described as a process changing over time [8], where the initial situation often entails being thrown into a changed role with new responsibilities but without enough information [9]. Experiences of loss of independence and inability to plan for the family’s daily life and future have been portrayed by family members, leading to feelings of being burdened, anxious, insecure, frustrated, and fatigued [9,10]. In a Swedish report, 45% of family members stated that the stroke survivor was dependent on their help with all or parts of daily activities. Furthermore, 9% of family members reported that they could not pursue their leisure interests, and 9% of those in the working age group had quit their jobs or reduced their working hours to take on the role of informal caregiver [11]. The report also revealed evident shortcomings in the support family members experienced receiving from the health care system.

Family members’ experiences of being involved in a client-centered activities of daily living (CADL) intervention for stroke rehabilitation has been explored [8]. Although the CADL intervention [12] did not specifically address family members (in this case, spouses), they described their involvement in the rehabilitation process. Family members experienced the rehabilitation process as twofold, encompassing both the aspects of caring for their partner and regaining their former roles and habits. In this strive, family members expressed feeling encouraged by their partners with stroke to spend time pursuing their own interests, which reduced their level of worry and fatigue [8]. The reciprocity in the interaction between the spouses and stroke survivors made it possible for spouses to continue being active while adjusting to a new life situation. This illustrates how interdependency can form a basis for resuming daily activities and accentuates the need to explore experiences and roles of family members in stroke rehabilitation [8].

The development of rehabilitation programs targeting both stroke survivors and their close network has been suggested [12-14] and can be a way of responding to the European Care Strategy for caregivers and care receivers [15]. Interventions with a focus on information, education about stroke, and problem-solving appear to benefit the relatives of stroke survivors [16], and combining this with a focus on activities can be favorable [16].

The role of digital health in developing rehabilitation programs is being increasingly discussed. Digital rehabilitation has been proven effective and applicable in various areas, including the improvement of motor and cognitive functions, mood, and daily activities [17,18]. The World Health Organization has suggested digital health as an umbrella term encompassing provision models for health and rehabilitation that use information and communication technology (ICT) [19]. ICT aims to facilitate communication between people and health care providers and can be achieved through various technological solutions, such as phones, computers, and tablets [20]. Digitalization is progressing rapidly, and according to Statistics Sweden, 84% of 65- to 74-year-olds and 62% of 75- to 85-year-olds used smartphones and apps in 2023 [21]. The use of ICT in rehabilitation after stroke is increasing, and there is a rapid development of apps as aids and as training tools [20]. However, cognitive, communicative, fine motor, and memory impairment connected to stroke can lead to challenges in handling technology [22]. To be successful, ICT-based interventions, therefore, need to be integrated into rehabilitation programs, including an educational component for caregivers paired with technical support [17].

The importance of investigating how the transition to digital health affects the close network around stroke survivors has been highlighted [23]. Furthermore, conducting qualitative studies on the need for support in the dyad of stroke survivor and family member has been emphasized as a key to evaluating intervention benefits over time [17]. This study explores family members’ experiences of an ICT-supported intervention, F@ce 2.0, that was integrated into rehabilitation for stroke survivors.

F@ce 2.0 is a person-centered, ICT-supported, team-based 8-week intervention for home-dwelling stroke survivors [24]. F@ce 2.0 has been developed through an iterative process in both Sweden and Uganda [25,26]. The intervention targets stroke survivors, aiming to improve their performance in daily activities and enhance their participation in everyday life. The rehabilitation team supports participants receiving F@ce 2.0 in formulating 3 goals related to activities they need and want to be able to perform in everyday life, as well as in finding strategies for accomplishing these activities. Reminders to perform the activities are sent through SMS text messaging each morning. Later in the day, participants receive a new SMS text message asking them to estimate how well the activities were carried out during the day. F@ce 2.0 has been evaluated in a full-scale, nonrandomized, controlled trial (trial registration NCT043511789; manuscript under review). In addition to the ongoing effectiveness study, there is a need to explore how the intervention was experienced by family members [26]. In a previous study, family members of stroke survivors receiving F@ce in Uganda expressed having a challenging everyday life with numerous responsibilities as well as financial strain because of having to give up work. Still, they felt supported by the F@ce intervention as it provided information on stroke and advice on training [27]. The experiences of family members of stroke survivors who received F@ce 2.0 in Sweden are not yet known and would add insights needed for further development of the intervention and the ICT support, taking family members’ need for support as well as the need for support within the dyad into consideration.

### Aim

This study aims to describe family members’ experiences of the F@ce 2.0 intervention from the perspective of being a caregiver to a stroke survivor.

## Methods

### Design

This study has a qualitative design and is based on semistructured individual interviews with family members of stroke survivors who participated in F@ce 2.0 as a complement to usual rehabilitation after their stroke. Furthermore, to describe participant characteristics, structured questionnaires and assessment instruments were used.

### Study Context

The study is part of an ongoing research project “F@ce 2.0 - Implementation and evaluation of a global model for a person-centered, ICT- and team-based rehabilitation intervention for people who have had stroke” at Karolinska Institutet. The aim is to evaluate the effect of integrating the F@ce 2.0 intervention in ordinary intervention, that is, if there are differences between the intervention group receiving F@ce 2.0 compared with those receiving usual intervention (controls). A total of 12 Swedish home rehabilitation teams, operating in both rural and urban settings, participated in the study, with 5 serving as control teams and 7 delivering the intervention [24].

### Participants

Participants were recruited among family members of stroke survivors receiving F@ce 2.0. Recruitment of stroke survivors was carried out by the rehabilitation teams participating in the effects study (manuscript under review). Within the F@ce 2.0 project, 100 stroke survivors were recruited into either the intervention group (n=45) or the control group (n=55) and were subsequently asked to identify a family member who could participate in the study. Family members were then contacted by a researcher who gave them more information about the study. If willing to participate, they also received written information along with a consent form. The total number of family members who accepted participation in the study was 38, with 20 in the intervention group and 18 in the control group. Recruitment of family members for this study started 9 months into the project, at which point 13 family members were already included in the intervention group. Of the 7 family members invited after this point, all agreed to participate in this study, resulting in a total of 7 participants: 4 husbands, wives, or partners, and 3 children.

Within the larger research program evaluating F@ce 2.0, additional data on family members’ situation was collected. To provide a more detailed background of the participants in this study, selected data is provided. The perceived burden after the 8-week intervention had ended was assessed using the Caregiver Burden Scale [28]. The scale ranges from 22 to 88, with a higher score indicating a more significant burden. Overall life satisfaction was reflected by the answer to the first question in the Life Satisfaction checklist [29], which uses a 6-graded ordinal scale from 1 (very dissatisfied) to 6 (very satisfied). The scale was dichotomized, and scores of 5‐6 were considered indicative of being “satisfied.” To give a background on the stroke survivors, stroke severity and perceived recovery are presented. Stroke severity was rated using the Barthel Index [30] (score range: 0‐100), where scores of 0‐49 points was considered severe to moderate, 50‐94 points was considered mild, and 95‐100 points was considered very mild [31]. Perceived recovery since the onset of stroke was rated using the visual analogue scale from the Stroke Impact Scale [32], ranging from 0 (no recovery) to 100 (full recovery). The characteristics of the participants and the stroke survivors are presented in Table 1, where the names are fictitious to maintain participant confidentiality.

**Table 1.** Characteristics of the participants and stroke survivors.

Name of family member	Relationship to person with stroke	Age (years)	Housing situation	Caregiver Burden Scale total score when intervention ended^a^	Family member response to LiSat-11 (life as a whole) at end of intervention^b^	Estimation of provision of help (0‐100)^c^	Gender of person with stroke	Person with stroke’s age (years)	Stroke severity at inclusion (Barthel index)	SIS^d^ 3.0 self-reported stroke recovery at inclusion
Christos	Son	36	Partly cohabiting	32	Unsatisfied	12	Man	71	Very mild	Missing
Farida	Daughter	28	Cohabiting	58	Unsatisfied	50	Woman	65	Very mild	40
Aino	Wife	87	Cohabiting	38	Satisfied	34	Man	93	Mild	60
Sara	Daughter	54	Living separately	55	Satisfied	50	Woman	86	Mild	90
Christina	Wife	86	Cohabiting	51	Unsatisfied	79	Man	89	Moderate to severe	20
Lennart	Husband	68	Cohabiting	33	Satisfied	70	Woman	72	Mild	30
Jan	Partner	84	Cohabiting	36	Satisfied	22	Woman	77	Very mild	50

a Caregiver Burden Scale consists of 22 items rated on a scale from 1 to 4. Score range: 22-88.

b LiSat-11: Life-Satisfaction Questionnaire-11; a rating of 5 or 6 is considered to indicate satisfaction.

c Participants’ self-rated estimation of the degree of assistance given to the stroke survivor (0‐100; 0=not at all, 100=to a large degree).

d SIS: Stroke Impact Scale; subscale for global rating of perceived recovery (0-100).

### Research Team

The research team consisted of 2 experienced researchers in both qualitative and quantitative research (GE and SG), 1 doctoral student (KS), and 1 clinician (ME), all of whom identified as female. All researchers had extensive clinical experience in stroke rehabilitation. GE, SG, and ME are all occupational therapists. KS is a speech-language pathologist. All researchers had in-depth knowledge of the F@ce 2.0 intervention.

### Ethical Considerations

The larger project of which this study is a part has ethical approvals from the Regional Ethical Review Board in Stockholm, Sweden (2013/1801-31, 2017/1420‐32), with a supplement (2020-01124) by the Swedish Ethical Review Authority. All participants received both verbal and written information about the study, including their right to withdraw their participation at any time. After receiving information and giving verbal consent, participants were asked to sign a written consent form. No compensation was offered. Aliases were used during data analysis to deidentify participants and data were accessible to the authors only.

### Data Collection

Semistructured interviews were conducted between January 2022 and December 2022 by an experienced clinician and researcher within the research group. The interviews were based on an interview guide and were performed on two occasions: when the 8-week intervention had concluded and again 4 months later (ie, 6 months after the intervention began). The interview guide was focused on 2 themes: perceptions of the life of the stroke survivors and the situation of being a family member to a stroke survivor. The first theme included questions such as “Can you recall how it was when your family member started the F@ce 2.0 intervention?” and “What is your perception of the SMS reminders?”. The second theme included questions such as “Are there activities that you used to do but no longer do?” and “Can you tell me how you have helped or supported a family member during the F@ce 2.0 intervention?” The second interview aimed at capturing the continued rehabilitation process after the intervention as well as experiences of the intervention over time. To address the issues that had emerged in the first interviews, specific follow-up questions were added to the interview guide before the second interview [33]. Of the 13 interviews, seven were conducted after 8 weeks. A total of 6 of the participants took part in a follow-up interview while one was present only for the first interview and then discontinued his participation due to lack of time.

Before the qualitative interview was conducted, the participants answered a questionnaire based on established assessment instruments. The interviews, both the quantitative part and the open-ended interviews, were conducted by an experienced researcher in the research group. The interviews were conducted by telephone due to both geographic distance and restrictions related to the COVID-19 pandemic, and were recorded and coded for deidentification before being stored in a secure database. The interviews were transcribed verbatim, and on occasions where words were difficult to ascertain, this was noted in the transcribed text. Field notes written down during the interviews were discussed in the author group during analysis to deepen the understanding of the context.

### Data Analysis

The analysis method chosen was a qualitative inductive content analysis, according to Graneheim and Lundman [34]. The analysis was planned to be kept as close to the text as possible, with the manifest content as the basis [34]. Initial coding was carried out by ME and later discussed with GE. As a first step, the transcribed texts from the interviews conducted at 8 weeks were read in their entirety several times to gain an understanding of the text. During this step, thoughts and reflections about the interviews were written down in a separate document. Parts of the text that were considered to have extra weight and corresponded to the study aim were marked. The second step involved extracting meaning units from the text [34]. This was done by using a scheme where the meaning units were condensed into shorter paragraphs, which were then coded (see Table 2) [34]. This part was kept as close to the text as possible. When the codes were set, the author went back and adjusted so that the code fitted into the context from which the meaning unit emanated, according to the process of working back and forth between the different parts of the analysis [34].

The codes from the different interviews were compared to discover if there were any similarities and differences. The next step after coding entailed categorizing codes based on their descriptions of the content on a more abstract level [34]. After analyzing the interviews conducted at 8 weeks, the same procedure was followed for the interviews conducted after 6 months, using a separate coding document. In this analysis, the experiences among the participants after the intervention had ended deepened the content of the previously developed categories, but no new categories were created. At this stage, KS entered the research process after having read through all interviews.

**Table 2.** Example of meaning units, condensed meaning units, and codes.

Meaning unit	Condensed meaning units	Codes
*Yes it’s a lot very much.. She has trusted them almost a little more than she has trusted me to do things like walk without a walker and try it she would never have done with me if they hadn’t been here, no she trusts them a little more because they were used to this kind of thing.* [Lennart, husband]	It’s a lot, she has trusted them more than me to do things like walk without a walker. She would never have done that with me. She trusts them more because they are used to this kind of thing.	Positive to get support from the team.
*It’s a really positive thing that he himself is kind of reminded because I think he sometimes forgets to do it but.* [Farida, daughter]	Positive that he himself is reminded, because I think he forgets.	SMS text messaging made a difference.
*This has kind of felt like some kind of step one as well.. This is like the beginning of something that can be or will surely be even better.* [Christos, son]	This has felt like the beginning of something that can be good.	The technology does not feel fully developed.

## Results

### Overview

**Figure 1.**
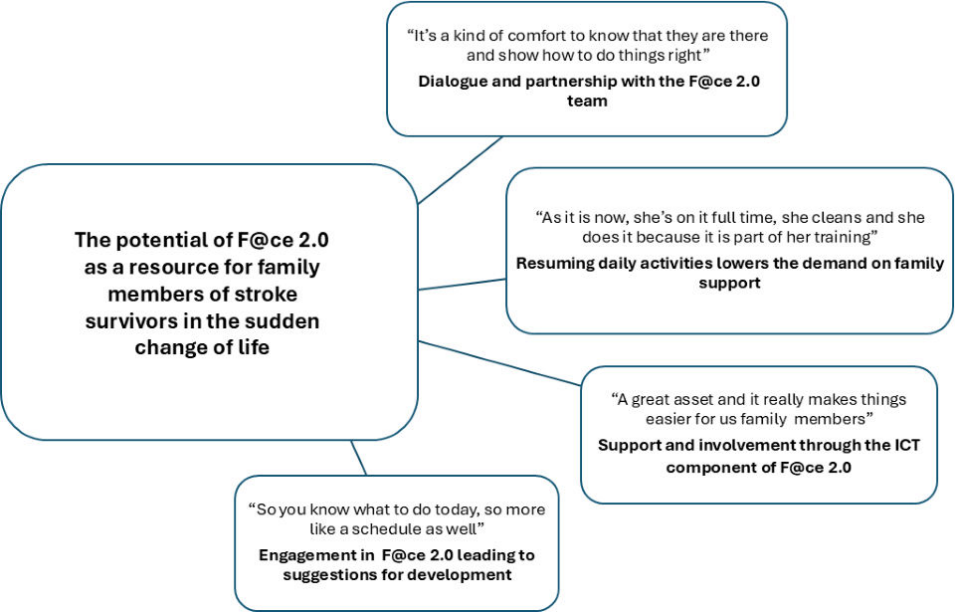
Overarching theme and categories.

The analysis resulted in an overarching theme: the potential of F@ce 2.0 as a support for family members of stroke survivors in the sudden change of life. This theme was developed against the backdrop of participants’ descriptions of life disruption invoked by the stroke as well as their perspectives on the need for support. A total of 4 categories formed the basis for this theme: dialogue and partnership with the F@ce 2.0 team, resuming daily activities lowers the demand on family support, support and involvement through the ICT component of F@ce 2.0, and engagement in F@ce 2.0 leading to suggestions for development (see Figure 1).

### Potential of F@ce 2.0 as a Support for Family Members of Stroke Survivors in the Sudden Change of Life

The study participants depicted profound life changes. Several of them had altered their habits and daily activities in a way that was described as undesirable but necessary and could be linked to a modification of roles related to living with a stroke survivor participating in rehabilitation. Both the onset of stroke in general and the participant’s involvement in the F@ce 2.0 intervention influenced these changes. Involvement in F@ce 2.0 could lead to changes that make everyday life more compatible with home visits by the rehabilitation team or with self-training of activity goals set up by the stroke survivor. A participant, for instance, mentioned getting up much earlier than usual to be ready for the teams’ visits, while others described adjusting their work hours to be there for their family members.

### Dialogue and Partnership With the F@ce 2.0 Team

Experiences of receiving support from the team varied among participants, but most described being pleasantly surprised by the support offered. They particularly appreciated it when the team took responsibility for the rehabilitation and ensured that follow-up appointments were scheduled. Positive experiences of the different professions in the team were narrated, along with the view that they, as family members, were seen and supported by the team.

Because we had so many questions as well because we wondered so much. Or I wondered so much. Even though there is so much to read, I still thought it was good to have a person you could bounce thoughts and ideas with regarding the training and the relationship to food.

[Christos, son]

Participants talked about anxiety connected to their family member having a stroke. An example is Christina (wife), who expressed that “It’s so silly, but I still have a bit of a hard time leaving him alone here at home. I know he’ll be fine, but the thought of him lying here alone...” This anxiety was described as decreasing when improvements were seen and when the stroke survivor received support from the team on how to act in different situations. Anxiety was discussed as a factor both for family members and stroke survivors, and participants highlighted that there sometimes was a need to have this as the initial focus of rehabilitation, creating a foundation for the rest of the process. Family members described how the presence of the team invoked a sense of safety in the overwhelming time after a stroke when they often felt insecure. The team’s engagement in discussion with the stroke survivor on how to perform activities in new ways, as well as leaving written instructions, was appreciated, for instance, by Christos (son), who stated, “It’s a kind of comfort to know that they are there and show how to do things right.”

The meaning of having a rehabilitation team present was accentuated during the second interview, where some participants shared a concern that the stroke survivor’s performance of activities was decreasing and a fear that these changes could lead to various deteriorations. Participants associated this change with a reduction in both the quantity and quality of training when the team was not present to follow up. “That’s precisely why I think it’s become that way, that he doesn’t train as hard as when they’re here,” said Christina (wife).

### Resuming Daily Activities Lowers the Demand on Family Support

The focus on daily activities in F@ce 2.0 was described as supporting both the participants and the stroke survivors in resuming their activities over time. Participants sometimes felt insecure about what support to offer the stroke survivor and recounted that the team provided information and engaged the stroke survivor in discussions about various ways to perform activities. In relation to this, setting goals for daily activities was discussed as one of the strengths of the F@ce 2.0 intervention. Christina (wife) expressed that “Just the fact that you have three goals, it is enough and that are not insurmountable, so you notice that it can be done...it was great.”

The participants described how the stroke survivor used daily activities as a form of training during the intervention, depicting how this made the stroke survivor more independent and able to practice without family members present to assist. “As it is now, she on it full time, she cleans and she does it because it is part of her training,” said Jan (partner).

The impact on the participants’ activities was described as most substantial in the first months after their family member had a stroke. With time and rehabilitation, participants felt that they had been able to resume more of their previous activities.

*The way we've had it, I've stopped right away, I had some courses and stuff like that that I stopped doing because I didn't want to leave him alone. But then I will take it up again this fall, absolutely*.

[Christina, wife]

### Support and Involvement Through the ICT Component of F@ce 2.0

The ICT component of F@ce 2.0, specifically daily goal reminders and ratings of goal achievement, was initially directed towards stroke survivors but appears to have the potential to support family members as well. Participants expressed wishes to be there for the stroke survivor and depicted how the daily reminder in that sense became relevant for both parties. Christina (wife) said, “It’s the thing with this reminder that first came in the morning… Then I only had to read to him and then it said that he should remember to do this and that and that....”

The text messages were also described as alleviating since they meant that participants did not have to be responsible for reminding the stroke survivor about the intervention goals and related activities. Being the one coming with constant reminders affected the relationship negatively as illustrated by Farida (daughter), “In that way, it’s also good that you don’t have to be the one who nags like… that you don’t have to be that character.” In relation to this she stated that the reminders were “a great asset and really makes things easier for us family members.”

Participants further described the SMS text messaging as a way for the stroke survivor to invite family members to be engaged and involved in the intervention. Sara (daughter) portrayed how her mother used SMS text messaging to engage her family in rehabilitation, “...and then she also sort of turned to us children and grandchildren and showed [the ICT part of the intervention] and thinks it’s exciting.”

Some participants depicted a deeper involvement that emanated from the stroke survivor needing support in handling the mobile phone. To overcome difficulties handling technology, the SMSs were sent to the family member instead of to the stroke survivor. This commitment was characterized as leading to shared reflection between the participant and the stroke survivor.

*It’s probably the understanding he has himself... he actually has that because we’ve both discussed and seen how things have gone and it’s also... thanks to the SMSs this follow-up that happens that is done*...

[Christos, son]

Participants viewed the commitment as something natural, connected to the needs of the stroke survivor. Some participants described how the intervention strengthened their relationship, despite initial apprehension.

*Perhaps you could say that it would potentially be annoying, maybe it would be something negative, but it’s absolutely nothing like that, I wouldn’t say that, it’s only positive.*

[Christos, son]

Participants outlined differences in involvement in F@ce 2.0 over time. They found that the initial phase required a greater commitment, which could later be reduced and ultimately perhaps ended.

*At the end here I was able to sit in another room and that was just fine... X [name] looked at me a little bit when she was going to answer questions and then I thought no, it’s better that you do it yourself*.

[Lennart, husband]

### Engagement in F@ce 2.0 Leading to Suggestions for Development

Engagement in the intervention led to reflections on the need to further develop it, particularly in terms of technology. Participants considered the intervention to be a good basis for rehabilitation support but identified several ways in which the ICT component could be improved. An aspect highlighted was the need to upgrade the goal ratings sent each afternoon. Although these questions were based on individual goals, participants perceived them as standardized and reported frustration because the goals were not updated when they proved too challenging or when they had been achieved. Participants reported that the stroke survivor became irritated when the questions remained the same all the time, without any development.

Participants also expressed a wish for a more significant part of the intervention to be digitalized. As Chrisos (son) expressed it “There is room for improvement, like more digitalization.” Family members’ experience was that after a stroke and in connection with rehabilitation, the health care system delivers a lot of information. To address this, there was a desire to have everything collected in one place, such as an app.

That way, you know what to do today, so it’s more like a schedule as well.

[Christos, son]

Participants also suggested making more use of video calls to facilitate communication with people who, for example, have aphasia. Video calls could also make it easier for the family members to participate in conversations with the rehabilitation team, even if they were not at home during team visits.

## Discussion

This qualitative study revealed that although the F@ce 2.0 intervention is primarily focused on stroke survivors, it also has the potential to support family members in adapting to life after stroke. The potential of F@ce 2.0 as support for family members was discussed by participants in terms of the meaning of the team’s presence, the focus on daily activities, and the support provided through ICT. Participants also offered suggestions on how to develop the F@ce 2.0 intervention further.

Experiences shared by participants in this study align with previous research, which portrays the effects of stroke on the entire family [35]. Participants depicted how the stroke survivors’ reduced independence led to a change in family roles. Although acting as a support person or carer was seen as a natural part of the relationship, it affected daily activities in several ways. Participants discussed changing habits and adapting their work situation to fulfill this new role. These findings align with several previous studies [14], underscoring the importance of not only directing rehabilitation towards stroke survivors but also of including the individuals around them.

Another topic in the interviews was feelings of anxiety related to the sudden change in life situation and uncertainties about the future. Anxiety among family members of people with stroke has been identified as a predictor of perceived caregiver burden [36], and addressing this should, therefore, be a key issue in rehabilitation. Participants in this study expressed how these feelings subsided when contact with the rehabilitation team was initiated. One aspect highlighted was the relief they felt when the team took responsibility for planning rehabilitation and booking home visits. Family members often find the transition from a more intensive rehabilitation service to community rehabilitation unsettling due to the decreased support [37]. Having a plan for rehabilitation contacts may, therefore, be perceived as helpful, even if visits are less frequent.

The ICT component of the F@ce 2.0 intervention was designed to support stroke survivors’ achievement of person-centered goals by daily reminders and follow-ups [38]. In previous research, health care professionals have suggested that ICT could play a role in supporting family members of stroke survivors by providing information as well as a framework for rehabilitation [39]. This study supports the idea that ICT can help family members by offloading them the responsibility for keeping up the structure in rehabilitation. For some, the SMS text messaging also led to a collaboration with the stroke survivor. This engagement could come from difficulties handling the mobile phone, but also from the stroke survivor wishing to share rehabilitation goals and progress.

Although the interviews demonstrated how F@ce 2.0 can support family members of stroke survivors, the results suggest that further development of the intervention is necessary to better meet the needs of family members. Despite the F@ce 2.0 intervention being home-based, some participants described having had no contact at all with the rehabilitation team. This finding aligns with other research indicating a paradoxical relationship between health care professionals’ awareness of the importance of informing and involving the network and the lack of readiness within the rehabilitation system to do so [37,40]. Family members should be invited to participate in activities when the rehabilitation team is present in the home to ensure they feel safe and can provide adequate support in daily activities and training [40]. Family involvement is encouraged in the F@ce 2.0 intervention; however, the intervention lacks a clear structure for implementation. Previous research has illustrated that rehabilitation professionals other than medical social workers may lack confidence when it comes to identifying and working with the social network around stroke survivors [40]. Future research should focus on developing strategies to integrate family involvement into rehabilitation interventions in a way that makes it a natural part of the process.

The notion of applying a family-centered approach, including rethinking the role of the rehabilitation team in relation to the family, is not new [7]. When implementing the F@ce 2.0 intervention in a Ugandan context, it was crucial to apply this perspective because the notion of person-centeredness collided with the family-based culture [25]. A way to meet the needs of family members in the future health care system could be to use telehealth interventions specifically designed for informal caregivers and family members. Promising but mixed results regarding such interventions have been demonstrated, and further research is warranted [41,42]. Others have argued that, instead of creating specific interventions for family members, a more effective solution would be to incorporate family support into existing programs [10]. Participants in this study recognized the potential of the F@ce 2.0 intervention but felt that the technology was outdated and desired a more comprehensive ICT solution that included information on stroke and rehabilitation. A recent evaluation of a Danish eHealth app focusing on stroke rehabilitation revealed that family members of stroke survivors appreciated having information about stroke readily available in the app and used it to prepare for meetings [43]. Future development of the F@ce 2.0 intervention could include a module for family members, along with a structured approach to ensuring family involvement. Here, it is crucial to co-design the content with family members of stroke survivors to ensure that it is genuinely relevant to the users [44]. Previous research has identified stakeholder involvement as a key explanatory factor for the success of family-oriented eHealth interventions [41]. Interventions also need to be flexible and allow for personal adaptations, as family members’ needs differ both individually and over time [45].

The findings of this study should be interpreted within the specific context of the Swedish health care system and society, where most of the population uses smartphone technology daily. Furthermore, data collection was initiated at a later stage of the overall F@ce 2.0 project, leading to a limited number of family members being represented. The diverse contexts of the different health care regions, as well as the participants’ varied backgrounds, did, however, lead to a variety of experiences and perspectives that are relevant and essential for the future development of person-centered stroke rehabilitation. Due to the COVID-19 pandemic, all interviews were conducted by telephone. This may have resulted in a lower degree of engagement among the participants. Participants had never previously met the person conducting the interviews, which made it challenging to establish trust in the remote interview situation. An additional methodological caveat is that they followed directly on a structured interview where the data collector obtained participants’ answers to the quantitative surveys. From the participants’ perspective, this may have led to fatigue, as well as a sense of having already discussed some of the issues. Performing data collection in reverse order might have yielded richer material. The study, however, adopts an exploratory aim and does not claim to have identified all relevant perspectives possible. A strength of this paper is the active participation of GE, KS, and ME, who all read through the interviews and were well familiarized with the material in preparation for discussion during the analysis phase. All authors have extensive experience in rehabilitation.

More than 5 years have now passed since the World Health Assembly Resolution on Digital Health was unanimously accepted by all member states [19]. A multitude of digital health interventions for rehabilitation have been developed and tested worldwide [18]. This study contributes to previous research, which illustrates that both family members and stroke survivors are able and willing to use eHealth [17,18]. It is, therefore, time for the countries of the world to move beyond discussing these issues and start implementing the World Health Organization recommendations, including adequate governance, infrastructure, and training, to ensure that the benefits of e-health interventions become an integral part of future health care [19].

## Abbreviations

C-ADL: client-centered activities of daily living
ICT: information and communication technology
